# On the systematic position of *Collyricloides massanae* Vaucher, 1969 (Platyhelminthes: Digenea) with notes on distribution of this trematode species

**DOI:** 10.1007/s00436-015-4333-2

**Published:** 2015-02-01

**Authors:** Gerard Kanarek, Grzegorz Zaleśny, Agnieszka Czujkowska, Jiljí Sitko, Philip D. Harris

**Affiliations:** 1 Ornithological Station, Museum and Institute of Zoology, Polish Academy of Sciences, ul. Nadwiślańska 108, 80-680 Gdańsk, Poland; 2 Institute of Biology, Wrocław University of Environmental and Life Sciences, ul. Kożuchowska 5b, 51-631 Wrocław, Poland; 3 Warsaw Zoological Garden, ul. Ratuszowa 1/3, 03-461 Warszawa, Poland; 4 Comenius Museum, Horní nám. 7, 750 11 Přerov, Czech Republic; 5 Natural History Museum, University of Oslo, P.O. Box 1172, N-0562 Oslo, Norway

**Keywords:** Trematoda, Microphalloidea, Collyriclidae, *Collyricloides*, Molecular phylogeny

## Abstract

The systematic position of the *Collyricloides massanae*, a rare cyst-dwelling parasite, located on intestinal wall of European birds and rodents, have always been controversial. Based on newly obtained sequences of the 28 sDNA of *C. massanae* from avian and rodent host from Central Europe, and on the previously published sequences of several genera and families among Microphalloidea, we evaluate its taxonomic position and the phylogenetic relationships within the genera *Collyriclum* Kossack, 1911 and *Collyricloides* Vaucher, 1969 which form the family Collyriclidae Ward, 1917. In the cladogram, *C. massanae* appears among the Pleurogenidae, forming a clade with *Gyrabascus amphoraeformis* (Modlinger, 1930) and *Cortrema magnicaudata* (Bykhovskaya-Pavlovskaya, 1950). We reject the commonly accepted placement of *Collyricloides* as the sister genus to *Collyriclum* within the Collyriclidae. Besides, we present and discuss the unusual records of *C. massanae* in the bank vole *Myodes glareolus* from northeastern Poland.

## Introduction

The digenean *Collyriclum faba* is a moderately well-known parasite found in subcutaneous tissue cysts in a variety of bird species. The systematic position of this parasite has been doubtful for many years: Odhner (1914) placed the genus in his family Troglotrematidae, while Ward (1917) regarding the isolated position of *Collyriclum* among monostomes, created the new family Collyriclidae Ward, 1917. Validity of the family was confirmed by Harrah (1922), but many authors of this period (e.g., Baer 1932; Witenberg 1932; Wallace 1935) continued to include *Collyriclum* within the family Troglotrematidae. The Troglotrematide proposed by Odhner (1914) was redefined by Dollfus (1939), leaving *Collyriclum* as the only genus within the Collyriclidae, a family placed within the superfamily Troglotrematoidea by Yamaguti (1971). In the latest classification based exclusively on morphological criteria, the Collyriclidae was included within the superfamily Gorgoderoidea Loss, 1899 and consists of two monotypic genera *Collyriclum* Kossack, 1911 and *Collyricloides* Vaucher, 1969 (Blair and Barton 2008); the latter erected to accommodate a digenean from rodents which lives in internal cysts and strongly resembles *C. faba*, but which possesses two suckers. Recently published analyses of variable 18S and 28S rDNA of *C. faba* have reassessed the relationships of *Collyriclum* and have placed it unambiguously within the Microphalloidea (Heneberg and Literák 2013; Kanarek et al. 2014).

The position of the genus *Collyriclum* is unquestionable, but the status of *Collyricloides* is much more contentious. Although similar to *Collyriclum*, *Collyricloides massanae* was collected from intestinal cysts in the yellow-necked mouse *Apodemus flavicollis* from SW France and differs especially by the possession of a ventral sucker (Vaucher 1969), but also in several important details of the arrangement of the genital- and excretory pores, the bursa cirri, the seminal receptacle and the metraterm. Based on these differences, it seems unlikely that *Collyriclum* and *Collyricloides* are closely related, but to date, there has been no question as to their membership of the Collyriclidae. We hypothesize that the resemblance between the two genera is superficial and convergent, and results from similarities in cyst-dwelling habitat, rather than being evidence of real phylogenetic affinity. In the present work, we evaluate the phylogenetic relationships of *Collyriclum* and *Collyricloides* within the family Collyriclidae, and present new data on the geographical distribution of *Collyricloides*.

## Materials and methods

### Sampling protocols

Material of digeneans from intestinal cysts were collected by dissection from a male Eurasian wren *Troglodytes troglodytes*, admitted to the Rehabilitation Centre for Protected Birds, Warsaw Zoological Garden which subsequently died. Necropsy revealed five large cysts on the serosal surface of the intestine; adult digeneans were isolated from the cysts, washed in tap water, fixed, and stored in 70 % ethanol. Additionally, five specimens of *C. massanae* were isolated from the cysts located on the intestine of adult male of European robin *Erithacus rubecula*, found dead on 6 April 2014 near Zahlinice, 15 km to south to Přerov, Kroměříž District, Moravia, Czech Republic. Specimens were isolated, processed as above and identified according to original description (Vaucher 1969). Voucher specimens, stored in ethanol have been deposited in Polish Collection of Parasitic Helminths, Museum of Natural History, Wrocław, Poland, Coll. No. 144283 (specimens ex *T. troglodytes*) and 144284 (specimens ex *E. rubecula*). Further samples of *C. massanae* were derived from *Myodes glareolus* from Urwitałt forest, northeastern Poland, where the parasite has been sporadically found to occur. In this case, during fieldwork in 2008/2009, two *M. glareolus* (out of c. 1000 examined over 10 years) were found infected with *C. massanae*. In both cases, up to seven parasites were located in cysts attached to the intestinal wall.

### DNA extraction, amplification and sequencing

Total genomic DNA was extracted from six worms (two from *M. glareolus*, two from *T. troglodytes*, and two from *E. rubecula*) following the manufacturer’s protocol (DNeasy Blood and Tissue Kit, Qiagen, Germany). The 28S rDNA locus was amplified using primers: forward—dig12 (5′-AAG CAT ATC ACT AAG CGG-3′) and reverse—1500R (5′-GCT ATC CTG AGG GAA ACT TCG-3′) (Tkach et al. 2003). The ITS1-5.8S-ITS2 region was amplified with the following primers: NLF/NLR (5′-TTTGyACACACCGCCCGTCG-3′/5′-ATATGCTTAArTTCAGCGGGT-3′) (Van der Auwera et al. 1994). PCR reactions were performed in a total volume of 25 μl containing 3 μl of genomic DNA, 10 mM Tris–HCl, 50 mM KCl, 1.5 mM MgCl2, 200 μM of each dNTP, 150 pmol of each primer, and 2 units of Taq polymerase (EurX, Poland). The thermocycling profile was as follows: 95 °C/3 min—initial denaturation; 94 °C/30 s, 52 °C/30 s (28SrDNA) or 48 °C/30 s (ITS complex), 72 °C/90 s—40 cycles; 72 °C/7 min—final extension.

The amplification products were purified using QIAquick PCR purification Kit (Qiagen, Germany) and sequenced in both directions (Genomed S.A., Poland). The obtained sequences were deposited in GenBank under accession numbers KP682451 and KP682452.

### Alignment and phylogenetic analyses

In order to elucidate any homologies with previously deposited sequences in GenBank, we conducted a BLAST search (www.ncbi.nih.gov/BLAST). This analysis showed that the sequences of *C. massanae* were closest to representatives of the superfamily Microphalloidea; thus, in the alignment, we use previously published sequences of Microphalloidea (Table 1). Sequences were aligned using the MAFFT v.7 software (www.mafft.cbrc.jp/) with FFT-NS-1 option. The phylogenetic analysis was performed using Bayesian inference (BI) with the MrBayes ver. 2.01 software (Huelsenbeck and Ronquist 2001); sequences of *Fasciola hepatica* (AY222244) was chosen as an outgroup. Bayesian inference was employed using the following nucleotide substitution parameters: nst = 6, rates = invgamma, that correspond to a general time reversible model (GTR) including estimates of the proportion of invariant sites (I) and gamma distribution (G). Posterior probabilities were approximated over 1,000,000 generations, log-likelihood scoters plotted and only the final 75 % of trees were used to produce the consensus tree by setting the ‘burnin’ parameter at 250,000. This number of generation was sufficient because the standard deviation dropped below 0.01 at the end of the run.

**Table 1:** The list of digenean species used in this study, with the information on their hosts, GenBank accession number and reference

Parasite species	Host species	Reference	GenBank accession number
*Lecithodendrium linstowi*	*Nyctalus noctula*	Tkach et al. 2003	AF151919
*Ophiosacculus mehelyi*	*Eptesicus serotinus*	Tkach et al. 2003	AF480167
*Prosthodendrium chilostomum*	*Nyctalus noctula*	Tkach et al. 2003	AF151920
*Prosthodendrium hurkovae*	*Myotis daubentoni*	Tkach et al. 2003	AF151922
*Prosthodendrium longiforme*	*Myotis daubentoni*	Tkach et al. 2003	AF151921
*Prosthodendrium parvouterus*	*Miniopterus schreibersi*	Tkach et al. 2003	AY220617
*Pycnoporus heteroporus*	*Pipistrellus kuhli*	Tkach et al. 2003	AF151918
*Pycnoporus megacotyle*	*Pipistrellus kuhli*	Tkach et al. 2003	AF151917
*Gyrabascus amphoraeformis*	*Myotis daubentoni*	Tkach et al. 2003	AY220620
*Brandesia turgida*	*Rana lessonae*	Tkach et al. 2003	AY220622
*Candidotrema loossi*	*Rana ridibunda*	Tkach et al. 2003	AY220621
*Parabascus joannae*	*Myotis daubentoni*	Tkach et al. 2003	AY220619
*Parabascus duboisi*	*Myotis daubentoni*	Tkach et al. 2003	AY220618
*Parabascus semisquamosus*	*Pipistrellus kuhli*	Tkach et al. 2003	AF151923
*Pleurogenes claviger*	*Rana temporaria*	Tkach et al. 2003	AF151925
*Pleurogenoides medians*	*Rana lessonae*	Tkach et al. 2003	AF433670
*Prosotocus confusus*	*Rana lessonae*	Tkach et al. 2003	AY220623
*Prosthogonimus cuneatus*	*Sturnus vulgaris*	Tkach et al. 2003	AY220634
*Prosthogonimus ovatus*	*Pica pica*	Tkach et al. 2003	AF151928
*Prosthogonimus rarus*	*Anas querquedula*	Tkach et al. 2003	AY116869
*Cortrema magnicaudata*	*Hirudo rustica*	Kanarek et al. 2014	KJ700420
*Collyriclum faba*	*Sturnus vulgaris*	Heneberg and Literák 2013	JQ231122
*Collyricloides massanae*	*Erithacus rubecula*	This study	KP682451
*Maritrema heardi*	*Oryzomys palustris*	Tkach et al. 2003	AY220632
*Maritrema neomi*	*Neomys anomalus*	Tkach et al. 2003	AF151927
*Maritrema oocysta*	*Hydrobia ulvae*	Tkach et al. 2003	AY220630
*Maritrema arenaria*	Cirripedia	Tkach et al. 2003	AY220629
*Maritrema prosthometra*	*Oryzomys palustris*	Tkach et al. 2003	AY220631
*Maritrema subdolum*	*Tringa erythropus*	Tkach et al. 2003	AF151926
*Microphallus similis*	*Carcinus maenas*	Tkach et al. 2003	AY220625
*Microphallus triangulatus*	*Somateria mollissima*	Galaktionov et al. 2012	HM584139
*Microphallus basodactylophallus*	*Oryzomys palustris*	Tkach et al. 2003	AY220628
*Microphallus abortivus*	*Hydrobia ulvae*	Tkach et al. 2003	AY220626
*Microphallus primas*	*Hydrobia ulvae*	Tkach et al. 2003	AY220627

## Results

**Fig. 1:**
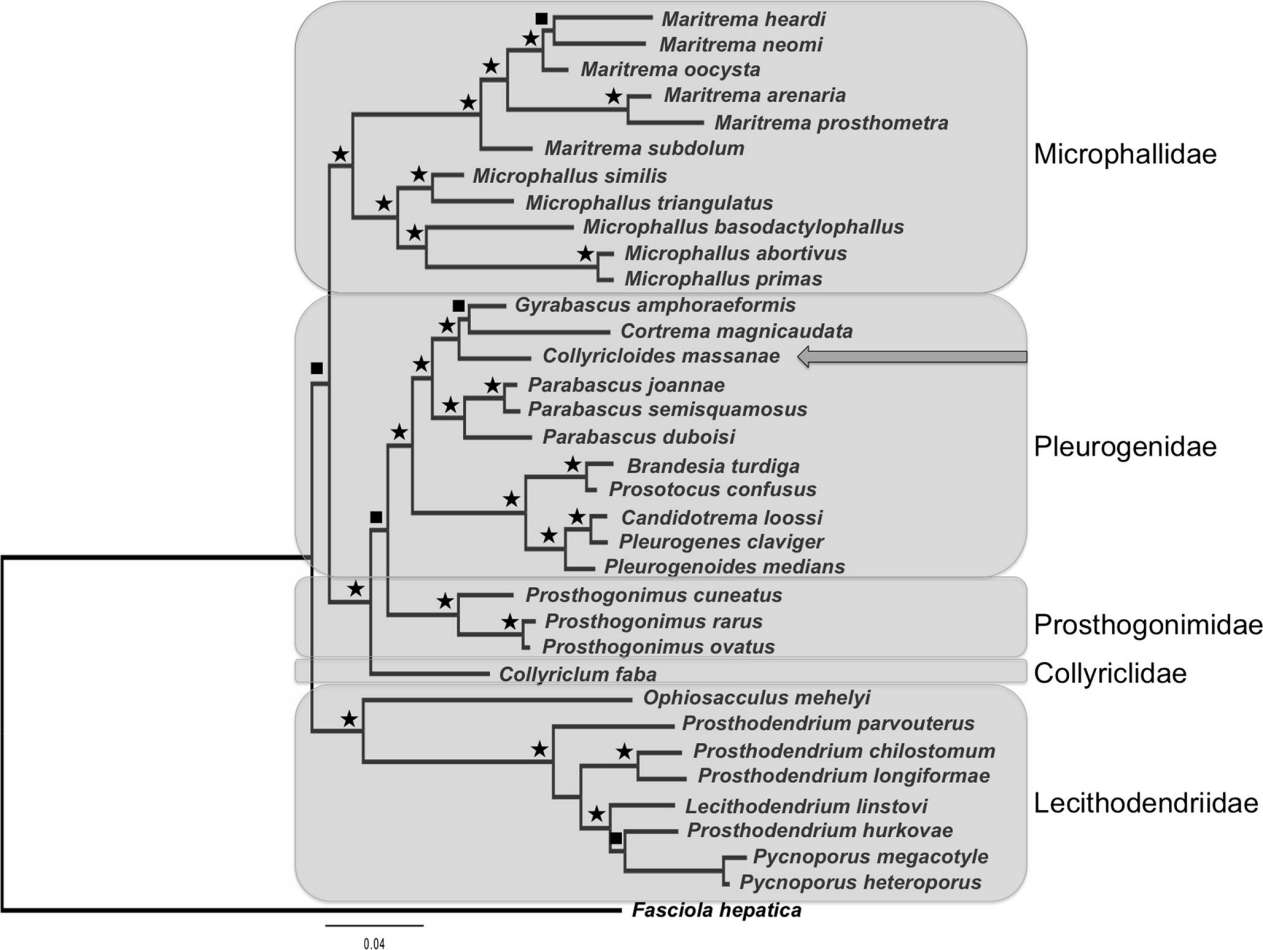
The phylogram resulting from Bayesian analysis of partial sequences of 28S rDNA gene. Posterior probabilities are expressed by the use of symbols: *star* (>90 % posterior probabilities) and *filled square* (>60 % posteriori probabilities). The *arrow* indicates the position of *Collyricloides massanae*

The final alignment of the lsrDNA fragment was 1275 bp long. The BI analysis resulted in a tree, with topology which strongly resembled the results from the previous phylogenetic study of Microphalloidea by Kanarek et al. (2014), except *C. massanae*, lacking in cited work. As in previous works, families Pleurogenidae, Prosthogonimidae and Collyriclidae formed 100 % supported clade, confirmed status of Collyriclidae as independent family within Microphalloidea (Fig. 1). *C. massanae* appeared amongst the Pleurogenidae and is closest to *Gyrabascus amphoraeformis* (Modlinger, 1930) (parasites of bats) and *Cortrema magnicaudata* (Bykhovskaya-Pavlovskaya, 1950) (parasites of birds). However, the clade uniting genera *Gyrabascus* and *Cortrema* is relatively weakly supported (65 %) which suggests that these genera may represent separate phylogenetic lineages. *Collyricloides*, *Cortrema* and *Gyrabascus* with three species from the genus *Parabascus* Looss, 1907 (parasites of bats) forming clade among Pleurogenidae uniting genera typical for warm-blooded animals. Sister branch of Pleurogenidae forming species characteristic from amphibians (genera *Brandesia* Stossich, 1899; *Candidotrem*a Dollfus, 1951; *Pleurogenes* Looss, 1896; *Pleurogenoides* Travassos, 1921 and *Prosotocus* Looss, 1899).

Additionally, as result of molecular analysis, we have obtained ITS complex sequences (ITS1, ITS2 and 5.8S rDNA) of *C. massanae* derived from three different host species. However, we did not observe the intraspecific variation in this region.

## Discussion

The present lsrDNA sequences-based phylogenetic analysis has demonstrated that genus *Collyricloides* belongs to the family Pleurogenidae within the superfamily Microphalloidea. Thus, the conventional view of *Collyricloides* as a sister genus to *Collyriclum* within the family Collyriclidae, held since the description of the genus by Vaucher (1969) and confirmed in the latest taxonomic arrangement of Blair and Barton (2008), is emphatically rejected. Based on molecular evidence, *Collyricloides* appears closest to bat and bird parasites *Gyrabascus* Macy, 1935 and *Cortrema* Tang, 1951, but remarkably, these three genera share few morphological features: only the pre-testicular ovary, the I-shaped excretory vesicle, a post- acetabular genital pore, and pre-acetabular vitellaria are common to all three. Both *Gyrabascus* and *Collyricloides* have virgulated xiphidocercariae developing in prosobranch snails (Burns 1961a and b; Schwarz 1981), but data regarding cercarial morphology of *Cortrema* (non-virgulated xiphidocercariae in pulmonate snails according to Tang and Tang (1981)) should be treated with caution (for details see Kanarek et al. 2014). Unlike *Cortrema* and *Gyrabascus*, which both lack a cirrus-sac and a visible pars prostatica and have a long, coiled seminal vesicle lying freely in parenchyma, *Collyricloides* possesses a well-developed cirrus-sac containing an internal seminal vesicle and a visible pars prostatica. Both *Collyricloides* and *Cortrema* possess a long Laurer’s canal, a seminal receptaculum, and long caeca, characters not seen in *Gyrabascus*. However, Schwarz (1981) contests the occurrence of bursa cirri in *C. massanae* (“Das Vas deferens führt zu einem kleinem schwach entwickeln Cirrus: ein Cirrusbeutel fehlt”), a detail noted in the original description (Vaucher 1969). Schwarz (1981) appears to have analysed morphology and anatomy of *C. massanae* without sectioning; in adult, whole mounts of this species, almost the entire body, is filled with eggs and anatomical details are almost impossible to discern. To sum up, the morphology of *Collyricloides*, *Cortrema*, and *Gyrabascus* is divergent, and while it is possible that these genera belong to different lineages amongst the Pleurogenidae, it is unambiguous that they are all pleurogenids and that *Collyricloides* is not part of the Collyriclidae.

At the same time that *Collyricloides* shows strong morphological differentiation from its closest relatives in the Pleurogenidae, it shows remarkable convergence with the morphology of *Collyriclum faba* from the unrelated Collyriclidae. This convergence has certainly confused most researchers who have compared these worms, including Vaucher (1969), who clearly believed the two forms to be closely related. The only characters noted as separating *Collyricloides* from *Collyriclum* were the differences in sucker number and differences in structure of the metraterm. However, as noted above, comparison of whole mounts is almost impossible because the eggs obscure all internal anatomy, and sections of the two forms have not been compared. The convergence is presumably due to the cyst-dwelling life style of both species, with numerous individuals living together within the cyst.

Another interesting issue concerns the geographical distribution of *C. massanae* and their host specificity. Natural infections of *C. massanae* have been detected several times in rodents (Jourdane and Triquell 1973; Mas-Coma and Feliu 1977; Schwarz 1981; Ribas et al. 2005) and in passeriform birds (Borgsteede and Smit 1980; Schwarz 1981; Sitko et al. 2006; Okulewicz et al. 2010) in Europe. *C. faba* has been recorded in a wide range of birds (mainly Passeriformes, but also Anseriformes, Galliformes, Charadriiformes, Coraciiformes, Piciformes) in Europe, Asia and Americas (for review see e.g., Bykhovskaya-Pavlovskaya and Khotenovskiĭ 1964; Stunkard 1971; Literák et al. 2003; Literák and Sitko 2006; Heneberg et al. 2011; Literák et al. 2011). All previous records of *C. massanae* in rodents (*Apodemus flavicollis*, *A. sylvaticus*) have been limited to mountainous areas of central (Schwarzwald) and southern (Pyrenees) Europe, while the occurrence of *C. massanae* in sedentary and short distance migrant birds (*Sturnus vulgaris*, *Sitta europea*, *Certhia familiaris*, *Turdus merula*) is mainly confined to northern Europe (see references above). Birds are much more mobile than rodents, and even for the sedentary or short distance migrants noted as hosts of *C. massanae*, migrations of several hundred kilometres are not extraordinary. Rodents, on the other hand, tend to have home ranges of a few hundred (rarely thousands) square meters (e.g., Kozakiewicz et al. 2007), and their helminth fauna predicts local ecological conditions. The life cycle of *C. massanae* can be assumed to be closely related with ecological conditions in mountainous areas of central and southern Europe, whereas findings in birds in Europe are more directly related to migratory behaviour. In this respect, the occurrence of *C. massanae* in *Myodes graeolus* from northern Poland is a complete surprise: this is the furthest north that this digenean has been found, some 1000 km north of all previous records. According to Schwarz (1981), the first intermediate host for *C. massanae* is the snail *Bythinella dunkeri*; invasive stages occur in insects which are related to stream ecosystems (Ephemeroptera, Plecoptera and Trichoptera). The genus *Bythinella* Moquin-Tandon, 1856 is distributed from the Iberian Peninsula to west Asia, and inhabit fast-flowing, cold, well-oxygenated waters and hypogean habitats (e.g., Falniowski et al. 1998). All species are alpine elements and glacial relicts (Falniowski 1987). Only a small number of about 90 described *Bythinella* species and subspecies (Prié and Bichain 2009) have been recorded from Poland, with their distribution limited to the southern parts of the country: *B. austriaca*, *B. cylindrica*, *B. zyvionteki*, *B. metarubra*, and *B. micherdzinskii* (Falniowski 1987). However, contemporary taxonomy of *Bythinella* is extremely complex with several controversies concerning the species distinctness (e.g., Bichain et al. 2007; Benke et al. 2011; Falniowski et al. 2012). In fact, it is impossible to distinguish the species without molecular analyses, consequently, the genus is often regarded as superspecies (Falniowski et al. 1998). At first sight, the Urwitałt forest represents an unpromising habitat for these snails, being an extensive, flat, cold, managed pine and birch forest dating to at least the early nineteenth century (Paziewska et al. 2010) and growing on sand and clay soils. However, *Bithyniella* is relatively resistant to desiccation and can be amphibious in behaviour (Falniowski 1987; Falniowski et al. 1998), and some isolated populations may exist within the Masurian Lakeland, rich in lakes and water courses with relatively cold and well-oxygenated water. In fact, the sample areas where the parasite was found in Urwitałt can flood in winter, and from one of the localities where *Collyricloides* was found, *Notocotylus* sp. was also found in *Myodes*, suggesting that the voles do have a semi-amphibious life style at some times of the year. The description of *Notocotylus malhami* from bank voles (Boyce et al. 2012) also indicates that this rodent can have a close relationship with amphibious molluscs in boggy or waterlogged ground. Nevertheless, it must be stressed that *C. massanae* was extremely rare at Urwitałt; this is the field site of the University of Warsaw/University of Nottingham long-term study of stability in bank vole helminth communities (Behnke et al. 2008a and b), and yet, the parasite has never been found in those surveys. At the same time, the parasite is cryptic; the cysts resemble extraneous pancreatic tissue and can be easily missed during dissection of freshly killed hosts, and so, it is possible that this digenean is actually much commoner in Europe than the existing records suggest. We also cannot exclude the possibility that the specificity of *C. massanae* to the snail intermediate hosts may be much broader than suggested by Schwarz (1981). These interesting issues will be examined in the forthcoming publications.
